# A qualitative study of Ebola survivors’ psychological experiences of evacuation, treatment and community reintegration: Lessons in holistic person-centred care from the 2022 outbreak in Uganda

**DOI:** 10.1371/journal.pmen.0000316

**Published:** 2025-06-23

**Authors:** Rwamahe Rutakumwa, Susan Ninsiima, Eddie Settuba, Leticia Kyohangirwe, Richard Stephen Mpango, Christine Tusiime, Joy Turyahabwa, Birthe Loa Knizek, Pontiano Kaleebu, Moffat Nyirenda, Hafsa Sentongo, Kenneth Kalani, Eugene Kinyanda

**Affiliations:** 1 Medical Research Council/Uganda Virus Research Institute & London School of Hygiene and Tropical Medicine Uganda Research Unit, Entebbe, Uganda; 2 Butabika National Referral Mental Hospital, Kampala, Uganda; 3 Makerere University Business School, Kampala, Uganda; 4 Department of Mental Health, Norwegian University of Science and Technology, Trondheim, Trøndelag, Norway; 5 Mental Health Division, Ministry of Health, Kampala, Uganda; 6 Department of Psychiatry, Makerere University, Kampala, Uganda; PLOS: Public Library of Science, UNITED KINGDOM OF GREAT BRITAIN AND NORTHERN IRELAND

## Abstract

The Ebola Virus Disease (EVD) is associated with significant mental health and psychosocial problems among patients/survivors. Effective strategies for addressing these problems necessitate an in-depth understanding of the sociocultural, economic and political context of the outbreaks, and survivors’ own experiences of the disease. Yet, such context and survivors’ experiences remain inadequately understood. We explore the pre- and post-diagnosis/treatment and post-recovery experiences of survivors of the 2022 EVD outbreak in Mubende District (Uganda) and draw lessons for better management of future outbreaks. A qualitative study was undertaken in Mubende District to explore the lived experiences of various categories of persons affected by the outbreak. Participants were sampled purposively. In-depth interviews were held with 15 EVD survivors. A thematic analysis of data was done. Our findings reveal that across the entire spectrum of their encounter with EVD, patients had a range of overwhelmingly stressful experiences that, according to the participants, took a toll on their mental health. These included stigma during evacuation and on return to their communities upon discharge, perceived inhuman treatment from health workers, lack of epidemic preparedness at the EVD facilities, witnessing frequent EVD deaths due to the EVD Treatment Unit’s open/unpartitioned ward, delayed removal of dead bodies from the shared ward, systemic lapses in health services and EVD-induced livelihoods challenges. In conclusion, EVD patients’/survivors’ experiences across the entire spectrum of their illness – from the period before diagnosis to their treatment at the EVD Treatment Unit and, later, their re-integration into their communities – are characterised with high levels of unattended, self-reported psychological distress. We propose a holistic person-centred approach to healthcare for EVD suspects, confirmed cases and survivors. Additionally, EVD education and sensitisation programs need to target health workers, among other population groups.

## Introduction

The Ebola virus can lead to life-threatening multiple system disease [1]. The largest and most fatal EVD outbreak since the virus was discovered in 1976 occurred in 2014–2016 in West Africa, with more than 28,600 cases and 11,325 deaths [2]. To-date Uganda has had seven EVD outbreaks [3]. The most recent outbreak in the country, which occurred in 2022, involved nine districts, with 142 confirmed cases, 55 confirmed deaths, 87 recoveries, 22 probable deaths (individuals who died before samples could be taken) and 19 healthcare workers, of whom seven died [4].

A lot of evidence has been generated on EVD, especially in the wake of the 2014–2016 West African outbreak [5–10]. We know, for example, that in addition to being a cause of high mortality and physical morbidity among patients and survivors, EVD is frequently associated with significant mental health and psychosocial problems among patients/survivors [11–16]. These problems often present in the form of formal mental health disorders of depression, anxiety disorders and post-traumatic stress disorder (PTSD) [11–13,16] and psychological problems of anger, suicidal tendencies, self-stigmatisation and sleep disorders [11,13]. Depression appears to be one of the most reported mental health disorders among EVD survivors. In Liberia, 75 percent of EVD survivors reported severe depressive symptoms within the first three months of recovery, although the rate reduced in the subsequent months [17]. In the DRC, 24.3 percent of EVD survivors reported depression, while 24.3 percent and 33 percent reported post-traumatic stress disorder and anxiety, respectively [16].

Despite this mounting evidence of prevalence and gravity of mental health problems among EVD survivors, the mental health of patients/survivors but also that of others affected by the disease remains inadequately attended to [12]. Likewise, only suboptimal attention has thus far been paid to experiences of EVD patients/survivors [18]. Yet, these experiences constitute a valuable source of firsthand evidence that would inform better responses to future outbreaks, at least insofar as characterising the traumatic events of an EVD encounter that might serve as precursors to the mental health problems exhibited later by EVD survivors. Additionally, given the novelty of the disease there is still room to learn from subsequent outbreaks [9,19], particularly about the “local social dynamics” with a view to informing “context-appropriate responses” to future outbreaks [19]. This is in light of the critical role of sociocultural, economic and political factors in influencing risk perception and behaviour, and the trajectory of EVD or other infectious disease outbreaks [10,20–22].

Our purpose in this paper, therefore, is to examine EVD survivors’ experiences with the disease prior to diagnosis, at the treatment unit and within their community after discharge from hospital, and to draw lessons from the 2022 outbreak in Uganda to inform evidence-based management of future outbreaks.

### Theoretical considerations

We adopted Critical Medical Anthropology (CMA) as our guiding theoretical framework. Developed in the late 1970s, CMA emerged essentially as a critique of conventional medical anthropology [23] on account of the latter’s exclusive concern with cultural determinants of health, and the consequent neglect of economic, political and social factors affecting health and health behaviour [24]. This exclusive concern with culture amounted to a “desocialisation of sickness and medicine,” whereby emphasis in healthcare is on “the primacy of the individual” or patient, and much less so on social determinants of health [25]. Yet, in adopting CMA we will, by extension, also be drawing on conventional medical anthropology’s legitimate critique of the biomedical model that erroneously assumes “disease to be fully accounted for by deviations from the norm of measurable biological (somatic) variables” [26]. It is this assumption that has justified a problematic focus in the treatment of disease on “biological and physical thinghood” with no regard for “the human relations embodied in symptoms, signs and therapy” [27].

Of seven key concepts that are identified as constituting CMA [24], we consider two to be especially relevant to our study. One of these is “disease,” which the author conceptualises as not only biological but also social. The social aspect of disease, according to the author, arises in the interpretations but also social relationships within family, community, social networks and religion, and how these shape beliefs, meaning systems and health behaviour. In our study, this theoretical grounding will be critical in illuminating the imperative for attention to the social mechanisms – that is, how the public processes and interprets a disease outbreak based on socially-constructed beliefs and meanings – that underlie the spread of disease. A recognition of such social mechanisms might be useful in, for example, understanding the public’s attitudes towards the EVD outbreak and, importantly, how these might have influenced the risk of catching EVD.

Another of CMA’s concepts that we consider relevant is “sufferer experience,” that is, an individual’s experience of sickness. Of note is that this experience is conceived as being shaped by not just the socially constructed meanings but also day-to-day economic and political dynamics [28,29]. Thus, there is need to particularly pay attention to politics and power relations and how these can shape the outcome of initiatives aimed at improving the human condition [30], in our case initiatives to control the EVD outbreak. This perspective seems to draw on the work of Foucault [31]. In his theorisation of the human body, and power and resistance, Foucault highlights the inherent agency of the subjects of power/ordinary citizens as expressed in the mundane “micro-resistances” or everyday acts of defiance (for instance, questioning norms and defying rules and expectations) against political or other forms of authority. We view sufferer experience, specifically its transcendence of the physiological experience to include the social and political aspects of the sufferer’s experience, as a useful conceptual tool for understanding EVD survivors’ interpretation of their health condition, their health seeking behaviour, their attitudes towards public health authorities and the trajectory of their illness. Accordingly, we propose that in situations of public health emergency, ordinary citizens will not always embrace official public health advice and, just as in normal situations, they will naturally engage in occasional acts of defiance against (public health) authority even if those acts constitute a threat to their own lives.

Another concept that we consider relevant for our study is “availability heuristic” [32]. Availability heuristic, otherwise referred to as availability bias, is a kind of cognitive bias or mental shortcut which aids a person in making a fast, though sometimes flawed, judgment [32]. A person employs the availability heuristic when they estimate the probability of an event by the ease with which examples of similar events in the past come to mind [32]. Thus, a person may quickly dismiss as implausible reports of an EVD outbreak in the community, even when officially confirmed, if they cannot think of any such outbreak in the community in the past. We consider availability heuristic useful for understanding Ebola risk perception and propose that when confronted with a risk as relatively infrequent as Ebola, some individuals may downplay its likelihood and continue engaging in high risk behaviour notwithstanding their awareness of its high fatality risk.

We draw on these theoretical perspectives to interpret and discuss our findings and to draw lessons for better management of future EVD outbreaks.

## Materials and methods

### Ethics statement

Ethics approval for this study was obtained from the Research and Ethics Committee of the Uganda Virus Research Institute (approval reference number GC/127/961), the Science and Ethical Committee of the London School of Hygiene and Tropical Medicine (reference number 29605), and the Uganda National Council for Science and Technology (reference number HS2870E). Written informed consent was obtained from each eligible participant. All the research procedures adhered to the relevant guidelines and regulations.

### Study design

A qualitative study was undertaken in Mubende District to explore the lived experiences of survivors of the 2022 EVD outbreak. The study was a sub-component of the broader study entitled: “Project to address the medium to long-term EBOLA associated psychological Distress and psychosocial problems in Mubende District in central Uganda” (Ebola+D study).

### Study setting

The study drew participants from all the nine sub-counties of Mubende District. The district is located in the Central Region of Uganda with an estimated population of 700,000, 65 percent of which living in rural areas.

### Study participants and sampling

Fifteen EVD survivors aged between 19 and 76 years were recruited for the study between September and December 2023, with eight of these being female. Participants were sampled purposively, initially from a list of patients attending Outpatient Departments at 6 health centres. Subsequently, snowball sampling was carried out in different communities within Mubende District. Participants were traced with the help of Village Health Teams spread across the nine sub-counties of Mubende District. To qualify for inclusion in the study, participants had to be 18 or more years of age, residing in Mubende at the time of the epidemic and have signed an informed consent.

### Data collection

Data were collected using a semi-structured topic guide initially developed based on the main research questions and iteratively refined during pre-test on the first four participants to ensure clarity, relevance and effectiveness of the questions. The guide was developed in English, translated to Luganda (the local language predominantly spoken in the study area) and back-translated to English to ensure the translated version accurately reflects the original meaning and cultural context. In-depth interviews were conducted with 15 EVD survivors. The sufficiency of this sample was determined when the data achieved the necessary depth and richness to address the research questions, and no new information was obtained with additional interviewing. Participants were interviewed by two research assistants who had undergone intensive training on the purpose of the study and the questions, as well as probing techniques to ensure depth and clarity of data, thereby assuring accurate representation of participants’ perspectives. The interviews were held at the participants’ health facility or any other venue of their choice usually within their community. The interviews, which were conducted in Luganda lasted 45–90 minutes and mainly elicited participants’ perspectives on their lived experiences with the EVD and their coping mechanisms.

### Data management and analysis

All interviews were audio-recorded with participant consent and transcribed verbatim in Luganda. These were then translated into English by the two well-trained research assistants who were proficient in both languages and had conducted the respective interview. To avoid data loss, each of the two research assistants back-translated the first of their counterpart’s translated scripts, and in both cases, the back-translations were compared to the original transcript and found to be accurate. An expert qualitative researcher conducted a thematic analysis, which was characterized by an iterative process initially involving reading all the interview transcripts as part of preliminary analysis. After gaining familiarity with the data, a detailed analysis of all the transcripts was undertaken, during which the data were broken down into various meaning units with respect to survivors’ lived experiences of the EVD infection, hospitalization and recovery. The meaning units were then linked together to form subthemes, from which key themes were abstracted. Using MS Excel, the key themes and subthemes were classified on a matrix, featuring relevant excerpts from participant narratives to illustrate the respective theme. The key themes emerging from this analysis included experiences prior to EVD diagnosis, experiences at the EVD Treatment Unit and experiences within the community following discharge from the ETU.

## Results

### Experiences prior to diagnosis

Experiences prior to diagnosis were encountered both within the community and at the Isolation Centre. In recounting the harrowing experience of an EVD infection, the survivors often narrated how it all started within the community. They talked about the excruciating uncertainty about a disease that was eluding diagnosis but whose symptoms were getting worse. They also exhibited misconceptions about the Ebola Virus Disease and dreaded the prospect of catching the virus as they associated it with sure death. In their narratives, survivors also notably pointed to psychosocial considerations in their decisions on where, when and how to (discreetly) seek care. These psychosocial considerations included how to navigate the potential ostracization by community members who did not want to catch the unknown but likely EVD infection. In this context of uncertainty and fear, the process leading to appropriate diagnosis and treatment of EVD patients was not straight. Rather, it was characterized by a sequence of decision points at which some critical and agonising decisions with implications for survival had to be made by the patient or their family members. These decisions, which sometimes involved reversal of an initially correct decision, usually centred on whether or not to seek services at the designated EVD diagnostic and treatment facility. Our analysis of data revealed two key drivers of such decisions.

One of these drivers were the EVD-sceptical private health workers. A striking observation from our data was that some health workers, particularly those who operated private health facilities, were expressing scepticism about the ongoing EVD outbreak, thereby undermining the official public health messaging about the outbreak. Some of these ventured further by promoting myths that, for example, top government officials were making a false alarm about the disease for their personal financial gain. Even more striking was that these health workers, who did not have personal protective equipment, appeared to be oblivious to the fatal risk they were undertaking by welcoming to their facilities and providing care to patients with symptoms that seemed to point to an EVD infection.

The experience of two survivors as narrated below may illustrate this point. One of these was a female survivor who initially had presented with suspicious symptoms at the regional referral facility designated for handling EVD but was still in denial about the possibility that she might have contracted the disease. She relates her experience of how she abandoned the referral facility supposedly to escape the risk of contracting EVD, eventually seeking care at a private health facility where doctors instantly ruled out EVD and offered to treat her illness. The doctors also expressed scepticism about the ongoing EVD outbreak and government’s awareness campaign.

*I [escaped from the referral where they were handling suspected and confirmed EVD cases and] walked home, after some time I developed a severe fever and then I opted to go to [name of clinic] because I never wanted to go back to the referral. Reaching the clinic the doctors told me that “those [are suspicious] government plans, we can treat that fever; that is not EVD.” So, they admitted me.* (Survivor # 15, Female)

The other survivor’s narrative spotlighted the experience of her EVD-infected sister who, for the same reasons as in the preceding case, was transferred from one facility to another but eventually died of EVD.

*She [sister] was even admitted at the hospital because of the asthma complication. So... when my brother-in-law came back from the market … he went to his friend’s hospital and came back home with him. He gave him the report from the doctor whom we saw first. On reading the report, this second doctor dismissed it …. He said “where could she have contracted EVD from? Those [EVD awareness campaigns] are just [suspicious] government plans. [Name of politician] just wants to steal our gold.” …. He [the doctor] … claimed that he will treat her, and that she will recover.* (Survivors # 10, Female)

In both the preceding two cases, the negative impact of health workers’ scepticism on the timing of patients’ access to appropriate care was clearly evident in the featured excerpts. But we also noted that this scepticism might have also reinforced patients’ own denial of EVD infection. For example, following the health workers’ unsuccessful efforts to treat the patients, we observed a tendency by the patients to make a series of attempts to access healthcare from facilities other than the designated EVD treatment facility. Survivor # 15, for instance, had the following to say as she described her next steps when the EVD-sceptical health worker did not help:

*I moved out of that clinic and went to Doctor [name of doctor]‘s clinic but she also works at the referral in the maternity ward. When she saw me with a suitcase and basin, she told me “you patients who come with such items we now fear to associate with you. You might be running from the referral.” …. So, I went back to the clinic I had started with in [name of place] …. On reaching the doctor’s clinic she asked me why I had taken all that time to go back for medication. Maybe she thought that I had healed, yet I was busy moving to different clinics.*

For her part, Survivor # 10 narrated:

*My sister said, ‘what kind of disease are they treating which is not getting cured?’ She decided to go to the pastor’s place who was her friend to pray for her …. As she was stepping down the motorcycle on her arrival she fell down and fainted. When the pastor saw this …they rushed her to [name of clinic]…. There was a doctor who examined her and said that she was injected with a drug which was inappropriate for her illness, and she got internal complications. The doctor said that they are going to start treating those wounds that she got inside her body.*

Drawing on Tversky and Kahneman’s [32] theoretical concept of availability heuristic, we view health workers’ scepticism about the EVD outbreak as having been informed by their observation that an EVD outbreak was improbable given (i) that there had never been any such outbreak in the community and (ii) the relative infrequency of EVD outbreaks in the country. More broadly, we interpret the denial of the EVD outbreak by the health workers, and of a potential EVD infection by the patients as a logical outcome of the socially constructed beliefs and meanings that proliferated at the height of the outbreak, as community members tried to make sense of the uncertain situation in which they were. As illustrated in the preceding two excerpts, such denial of EVD infection had the effect of further delaying vital access to appropriate EVD care, which in some cases might have been the cause of the patient’s death.

Aside from the EVD-sceptical private health workers, another driver of the patient’s/family’s decision on whether to seek services at the designated EVD diagnostic and treatment facilities was the fear of being stigmatized by community members for contracting a disease with such high infectivity and case fatality rate. This fear was so profound that patients were not only concerned about living with the stigma – by way of labelling/verbal abuse and social isolation – later as EVD survivors but also, in case they did not survive, the stigma of having died of EVD. Still, even when they made the agonising decision to seek care at the EVD Treatment Unit, concerns about stigmatization by community members sometimes weighed heavily on the timing of the patient’s transfer and the mode of transportation to the EVD facility. These concerns were on display in one of the cases, as illustrated below.

*[During evacuation] I regretted why I even called that ambulance because I wanted to walk to the hospital. I feared stigma from the community, talking about me that I died of EVD or I have been infected with EVD. I tried walking to the EVD Treatment Unit but I moved from the house to the main road and failed to cross the road because I had general body weakness, headache…. I had also taken time without eating because whatever I could eat would come out immediately. The ambulance came and picked me up [from the main road]. But it was like an alarm because everyone got out of their house to see what was going on as I was considered to have been infected with EVD, and after discharge, it was really hard for me to get back to the community. They would say: “that person got infected with EVD” ….* (Survivor # 11, Male)

We noted therefore that concerns about stigma constituted a key part of the EVD sufferer experience and had an important bearing on the sufferer’s illness trajectory and treatment outcomes. This is because the concerns bred indecision about seeking help, which in turn resulted in potentially fatal delays in accessing healthcare.

Beyond the community, patients suspected to have EVD had distressful experiences at the Isolation Centre, which served as a holding place for EVD suspects who were evacuated from the community and were awaiting diagnosis. These evacuees ranged from patients in a medical emergency to those acting out of caution and looking to be tested to rule out EVD after noticing suspicious symptoms. We observed from our analysis of data that patients’ experiences at the Isolation Centre revolved around perceived inhuman treatment of patients and concerns about the level of organisation of the Isolation Centre and its readiness to handle the EVD outbreak.

Perhaps one of the most stressful experiences patients encountered at the Isolation Centre was the perceived inhuman treatment from health workers. This was particularly with respect to the manner in which the health workers implemented established protocols for further management of suspected cases whose test results had confirmed an EVD infection. A case in point is one that involved a mother who was being held at the Centre with her three weeks old baby as the two awaited EVD test results. The patient narrates her unbearable agony arising from what she perceived as the abrupt and cruel separation from her newly born baby after their test results were found to be discordant.

*So, they took our blood samples that morning, and the results were brought at night, as we had dozed off. The doctor pulled my child from me. The baby was three weeks old, and he cried because he was scared from sleep. I too got scared…. Then the doctor told us to [prepare] and follow a certain doctor. I asked why … and she said, “your results are back and you are positive but the child [is] negative.” You cannot understand the stress I had at that time while they were taking my child who was three weeks old. He … would cry a lot and I was the only person who would calm him down…. As the child was crying, I told the doctor to at least allow that he goes after breastfeeding. The doctor replied: “are you stupid, we have told you that you are infected, would you also want to infect the child?”.... So, we went while I was crying. I could not even go back to my baby. I really felt grief and I cried. This sickness never hurt me like the way the intense emotional pain did.* (Survivor # 10, Female)

Besides the extreme emotional pain it occasioned on the patient, the act by health workers of taking the baby away in a manner that they did was remarkable insofar as it laid bare the inadequacy of the approach to healthcare that focuses on the human body largely to the exclusion of the patient’s psychological/emotional needs. We noted that notwithstanding the patient’s survival from EVD, the health workers’ disregard for the patient’s emotional health needs as evident in the way they separated her from her baby and responded to her request to breastfeed the baby might have negatively impacted her recovery trajectory, but also had the potential to jeopardise her chances of survival.

Apart from perceived inhuman treatment as presented above, our analysis of data revealed that patients’ experiences at the Isolation Centre also revolved around concerns about the level of organisation and epidemic preparedness. Participants specifically pointed out the lack of basic amenities, such as electric power/lighting and toilets, with the latter resulting in dangerous disposal of highly infectious waste. Relatedly and perhaps more importantly, they also described their discomfort with the practice at the Isolation Centre of holding all the suspected cases in a single room without regard to the risk of infection of EVD-negative suspected cases by those who had already contracted the disease. One of the surviving patients had the following to say:

*They were not prepared and for a person who was not infected it could be easier for the infected person to spread the disease to them…. where infected people are vomiting and having diarrhoea, that only could transmit the disease to them …. It did not have electricity and we would use our torch for light, we never had a toilet and they used to give us buckets for easing ourselves which we emptied in another big bucket. So [under those circumstances] … other people [could have] also been infected with the disease.* (Survivor # 9, Male)

### Experiences at the EVD treatment unit

Survivors shared diverse experiences at the EVD Treatment Unit (ETU). In some instances, these were positive. For example, several of them credited health workers for their professional and compassionate treatment of patients, and government for full coverage of costs of their medication and nutrition.

*They [health workers] did not segregate us, isolate or even discriminate us. They treated us [even] better than they treat their children…. The way we were having diarrhoea, if you were not having a bold heart you would not handle those patients but they handled us so well…. We got medication on time….* (Survivor # 7, Female)

*They [Government] treated us well because there was what to drink and what to eat, though we could not eat. But breakfast, lunch and supper were there; water, sodas, they used to bring…. Government used to bring them.* (Survivor # 2, Male)

However, our analysis of data revealed that narratives on survivors’ experiences at the ETU were for the most part negative. These mainly related to the ETU’s physical layout (the open concept) and the health services provided at the ETU.

### Experiences with the ETU’s physical layout (the open concept)

One of the patients’ key concerns at the EVD Treatment Unit related to the physical layout of the Unit, specifically the open concept whereby all EVD patients were admitted together in an open, unpartitioned large ward. In only a few cases, patients alluded to the positive aspects of this physical layout of the Unit in terms of enabling the willing recovering patients who were still in admission to help fill some service gaps. This included providing the very ill patients with assistance that did not require the expertise of a nurse or doctor.

*We used to call them [doctors] whenever there were patients in bad condition. But they used to take a long time to reach our unit, so that was our job, those [of us] who had some energy, because you would not wait for a doctor to help your fellow patient to wash yet you can wash for them, and you have some energy.* (Survivor # 10, Female)

Still, in most of their reflections on the experience at the ETU, survivors resented the open concept mainly for two reasons. The first was that it created an undesired situation where patients were witnessing frequent death all over the Unit. This caused considerable anxiety and emotional stress to the patients, as they were now perceiving imminence of their own death. Furthermore, for some patients this experience reportedly elicited what we view as maladaptive responses, such as loss of appetite and poor treatment adherence. This might have complicated the patients’ recovery path and, for other patients, could have contributed to their death.

*I feared even to sleep on that bed. What broke me was that I was very weak but some patients [who] were brought in and … were stronger than me… would last like an hour or few minutes and die. This caused me a lot of fear. I started losing appetite for food; even taking ORS was a problem…. Man, things were bad, and the worst part was seeing someone you have just been talking to a few minutes back … dying….* (Survivor # 13, Male)

The second reason why survivors resented the open concept was that no gender considerations were made in the admission of patients. As a result, there was a lack of privacy as women and men shared the same space in the ward. This was especially stressful when, as often happened, a patient needed to undress but was not comfortable in the presence of the opposite sex or, worse still, when a member of the opposite sex lost control of themselves and undressed or soiled their clothes.

*The bad thing I saw was mixing males and females in the same room. During that time, you could not undress because both genders were in the same room. There was a man who failed to walk, and he was crawling naked on his belly. He was covered with blood all over his body. He could crawl under my bed, and he shakes the bed. Sometimes it would move out of its position, yet I was on drip…. Mixing us [female and male] … was very bad.* (Survivor # 15, Female)

### Experiences with health services at the ETU

One aspect of health services at the ETU that elicited patients’ negative experiences was the nature of health services, insofar as the services were essentially oriented towards the patient’s physical recovery largely to the exclusion of their mental health. This absence of a holistic approach to healthcare was alluded to by the patients as they shared about their interactions with health workers but also with the services themselves. Two scenarios from the data may illustrate this finding. One of these related to a case of a patient, referred to earlier, who was dramatically separated from her three weeks old baby after the latter tested negative for EVD. We observed that the healthcare needs of this patient – who had remarkably disclosed that the intense emotional pain from being separated from her newly born baby was more traumatising than her EVD illness – clearly exceeded the physiological treatment of her EVD infection. She also needed care for psychological stress that, as evident in the following excerpt, was undermining her treatment adherence and could have confounded her overall recovery.

*The entire time my child was in the hospital I never got any sleep. Because every time I would hear him cry, I would get out [of the ward] and remind health workers that they promised to take care of my child and would ask them why he was crying. It was a rainy season, but I would stand by the wire mesh as it rained on me. Doctors would plead with me to get back inside the tent because I was ill. But I used to tell them that I’m not going to get away from the wire mesh until my child has kept quiet.… Every time I heard my baby cry, I would unplug the cannula from my body and go stand by the wire mesh.* (Survivor # 10)

Although the health workers evidently tried to address her psychological stress by comforting her as she agonised over her crying baby, it was noteworthy that no mental health support was available to her as part of the formal health services at the facility. The absence at the ETU of mental health services and specialists in such a highly stressful epidemic situation may be illustrative of the endemic shortage of mental health professionals. Nonetheless, it pointed to what theorists, as discussed earlier, have viewed as an emphasis in healthcare on biological and physical thinghood in the treatment of disease, resulting in the “desocialisation of sickness and medicine. The futility of such an approach to healthcare that does not pay attention to “sufferer experience” [24], in this case the patient’s social and emotional world, was evident in the patient’s interruption of her own EVD treatment whenever she felt that the welfare of her baby was at stake. Additionally, such interruption of treatment and her defiance of the health worker’s advice to return to the ward illustrated what has been characterised as the micro-resistances or everyday acts of defiance by subjects of power against authority even if those acts constitute a threat to their own lives. Our findings therefore indicate that inattention to patients’ social and emotional world, and the resultant maladaptive coping responses by the patients (that is, attempts to defeat their own treatment) amounted to a critical omission in the care for EVD patients, with a potentially negative bearing on patients’ treatment outcomes.

The other scenario illustrating absence of holistic care at the ETU was the delayed removal of dead bodies from the ward following death of EVD patients. We recognise that for a poorly resourced and ill-prepared healthcare system, prompt removal of dead bodies may have been a challenge given the usual staff shortages. Besides, such removal could only be safely done by staff equipped with personal protective wear, which may have not been sufficiently available. Even so, the delayed removal of dead bodies from an unpartitioned ward with many patients in admission depicted the health facility’s remarkable inattention to patient’s care experiences and expectations, and their mental health. Indeed, the gravity of this inattention was highlighted in the reported negative impact of such delay on the mental health of patients on the ward. Even as they attributed the delay to the frequent EVD fatalities that had led to normalisation of death, patients lamented about the stress of having to coexist with a dead body on the ward for an entire day.

*What I realized is that health workers reached a time when they were tired of the situation, and in case someone died it was not news to them. She died and spent the entire day [in the ward]…. It was not good! The thing that stressed me [the most] was for someone to die and the body is treated like anything that does not have value, [by] not picking these bodies in their required time.* (Survivor # 9, Male)

Another aspect of health services at the ETU that elicited patients’ negative experiences were the systemic lapses in health services at the Treatment Unit. These lapses were specifically in relation to the daily long stretches of time when no doctor or nurse or other health worker was deployed on the ward (ETU). Given the nature of EVD and its fast progression, these lapses, which were normally scheduled in the night, aroused tension among the patients who reportedly had to endure hours in anxiety as they dreaded a likely moment when they are in emergency need of help and there is no health worker on the ward to attend to them. It was clear that this lapse in services and the crisis it engendered was one of the most stressful experiences among patients, especially since the patients had come to associate nighttime with death on their ward.

*Maybe the sickness used to become aggressive in the night, yet the health workers used to stop at night…. And that is when the patients would cry, shout and get worse…. Because the illness gets tough at night … people die in the night not daytime…. The mistakes that were there were that they [doctors & nurses] were not working at night, stopping at 10pm. Then they go to sleep.… There was a time when seven patients died in one night. There was noise everywhere by the patients because when you see all your colleagues dying you get scared!!* (Survivor # 2, Male)

*These people [health workers] had a tendency of announcing that the next attendant is coming after like 5 or 6 hours but remember someone’s condition can worsen after just these other [health workers] have left…. but … if they send like two people just to be checking on patients’ condition, … [that would help].* (Survivor # 13, Male)

### Experiences within the community following discharge from ETU

#### Stigma.

As might be expected, stigma emerged as a key concern among survivors when they returned to their communities following discharge from the EVD Treatment Unit. In fact, our analysis of data shows that it was the major cause of survivors’ negative experiences within the community. The sources of stigma were varied, including what may be considered likely sources, namely community members. Survivors narrated about their negative experiences in their different encounters with community members, including avoiding physical contact with the survivor for fear of contacting the Ebola Virus Disease.

*What was distressing was a lot because they used to see you [the survivor] conversing and they are coming. Then they just turn and use another route as if they have not seen you; as if you have quarrelled [with them] …. I did not feel good about it….* (Survivor # 3, Female)

In other incidents, community members’ stigmatising behaviour seemed to be prompted not just by the fear of infection but also by downright cruelty.

*My home is along the road. So, sometimes as someone is passing by … they shout at me “Ebola man!” [In other instances] I would be walking on the road and someone greets me: “Ebola man how are you?”* (Survivor # 12, Male)

However, we also notably observed stigma from unlikely sources – institutional agents. These included health workers and school administrators whose community engagements or other actions had the effect of stigmatising the survivors or their family members. In some cases, this stigmatisation was inadvertent, having been the incidental outcome of well-meaning efforts by the agents to ensure survivors’ safe re-integration into the community. This was true in the case of health workers. In their community engagements, health workers from the EVD Treatment Unit had emphasised the importance of EVD survivors using condoms with their partners in the immediate aftermath of their discharge from the ETU, given that the virus may persist in semen long after male patients survive. But the unintended effect of this otherwise life-saving message was the stigmatisation of male survivors who were now being viewed by other community members as carriers of EVD, and therefore a threat to the community.

*They [community members] still think that we who suffered from EVD still have it. They have not believed that we no longer have it in our bodies. The health workers taught … us to use condoms with our wives [for the time being], but they later tested us and found that our bodies were EVD-free. However, the health workers did not tell the people, so they still think that we … have EVD.* (Survivor # 6, Male)

The other kind of stigma from institutional agents was direct, being triggered by the agents’ insensitive and unjustified decisions. Survivors repeatedly singled out schools as the source of such stigmatisation, which was usually exhibited in students being blocked by the school administration from attending school if their family members were known to have suffered from EVD.

*My children were stopped from going to school because of having someone in the family infected with EVD, I have two school-going children but when the school administrators got to know that I was hospitalized for having EVD, those children were discriminated against and stopped from going back to school. Even my father’s children were stopped. He ended up [transferring] them … to boarding [school].* (Survivor # 15, Female)

It was striking to observe that stigma was perpetuated not only by community members, who understandably might have acted out of ignorance, but also by schools which ordinarily would have been expected to have a good command of facts and to provide institutional opinion leadership on these.

#### EVD-induced livelihoods disruptions.

For several survivors, the reality of life following recovery from the EVD was difficult, as their livelihoods were severely disrupted by the disease. On returning to their community, they found nothing to harvest from their gardens, and their businesses had collapsed. In these circumstances, they did not seem to have any other option than to attempt to rebuild their livelihoods all over again. Yet, this was itself not realistic given that the survivors were only beginning to recover from the physically debilitating effects of the EVD infection and did not have the physical stamina to embark on this. In effect, the disease had escalated poverty and vulnerability among survivors and their families, which contributed to high levels of distress as the survivors were not able to provide for themselves and their families. It is in light of this that the survivors were counting on aid to address their livelihoods challenges.

*If they [health workers] had kept us on the diet that we were on [in hospital], we would have been ok. But you get to the village, and you have nothing to do because the three previous [planting] seasons we have not worked at all. Maybe they should give us money. The fact that am a man and I have a family, I have to spend on medication, the children want milk…. You must provide [for family]….* (Survivor # 6, Male)

## Discussion

In this paper we set out to examine EVD survivors’ experiences with the disease prior to diagnosis, at the treatment unit and within their community after discharge from hospital, and to draw lessons from the 2022 outbreak in Uganda to inform evidence-based management of future outbreaks. One notable observation from our findings was that survivors’ experiences across the entire spectrum of their illness – from the period before diagnosis to re-integration into their communities – had a lot to do with high levels of psychological stress. That an encounter with the highly fatal EVD was associated with high levels of psychological stress might ordinarily be expected. Indeed, participants narrated about the heavy toll of the disease in the form of extreme physical stress on their bodies and their excruciating fear of death from it, and the well-documented stigma of an EVD infection [12,18,33–36].

However, what was unexpected were the service- and infrastructure-related stressors of the EVD Treatment Unit. We viewed these stressors as broadly illustrating absence of holistic person-centred healthcare, defined as an approach to healthcare in which the health worker attends to the needs of the whole person as they relate to body, mind and spirit [37] and is focused on what the patient wants as a person [38]. Lack of person-centred healthcare was evident, for example, in the act by health workers of abruptly taking a baby away from the mother without preparing her for this eventuality or providing her with any form of counselling. This act might be excusable given that the health workers presumably were following institutional safety protocol in such situations of emergency, and that health workers in such a high case-fatality risk environment themselves arguably needed support to be able to serve patients to the latter’s expectations. Nevertheless, such abruptness by the health workers and the tense health worker—patient exchange that ensued during the process spotlighted the need for the healthcare system to pay attention to the patient as a whole person with needs that transcend mere physical illness to include dignity and understanding. This is consistent with Singer’s [24] call for attention to “the human relations embodied in … therapy,” and minding that the patient’s emotional state – itself a function of satisfaction with not just their own but also the wellbeing of loved ones – has an important bearing on their therapy outcomes.

Our study echoes previous research [39] in illuminating delayed removal of dead bodies from the EVD ward as a major source of psychological stress to patients on the ward. The significance of delayed removal of dead bodies in terms of its adverse impact on patients’ mental health may be better appreciated in view of the high cultural sensitivity around, and fear of, death and dead bodies in the African context [40]. We therefore view such delay as constituting a critical gap in service delivery, more so in the context of a highly fatal EVD epidemic, where a dead body on the ward may serve as a constant stressful reminder to other patients that their own death is next.

The finding that some health workers, specifically those in the private sector, were undermining the fight against EVD was remarkable. We observed that these were not just sceptical about the EVD outbreak but also promoted myths that top politicians were making a false alarm to justify a lockdown and secretly exploit the community’s natural resource wealth (gold). This finding lends credence to previous research [20–22] which has called for infectious disease outbreaks such as EVD to be viewed not only through the lens of biomedicine but also as social, economic and political events that necessitate inclusion of social, economic and political responses. The finding also validates those from previous studies [10,19] which have illuminated the need to pay close attention to the role of context or local social dynamics in the study of EVD outbreaks. This is because such attention carries the potential for discovery of new context-specific knowledge that may facilitate development of locally acceptable and more effective solutions. Indeed, to the best of our knowledge, our study is the first to have noted the direct involvement of health workers in active efforts to undermine the fight against EVD.

### Lessons

First, the healthcare system’s evident preoccupation with patients’ physical recovery and absence of mental health services in the midst of acute need, point to a critical need for holistic person-centred healthcare to EVD patients/survivors. In practice, such care would entail health worker responsiveness to the patient’s/survivors’ psychosocial and emotional needs and, at systemic level, the integration of mental health services and specialists in the planning and management of EVD and other infectious disease outbreaks. This will, among other things, help minimise re-traumatising of the victims, such as survivors who may have overcome the trauma of an EVD infection only to be met with fresh trauma (stigma) on returning to their communities. Adoption of person-centred care is further justified by its demonstrated positive impact on healthcare outcomes [41], including shorter hospital stays and better health-related quality of life [42] and reduced costs of care [43]. Second, strategies for better management of future EVD and any emerging infectious disease outbreaks that may have little or no history within the affected community need to pay attention to availability heuristic, a common heuristic in risk perception. This is because of its potential to generate public complacency towards the outbreak, more so when it influences the judgment of health workers who otherwise would be at the forefront in efforts to control the outbreak. In the same vein, the fact that some health workers were undermining the fight against EVD demonstrates the importance of not taking any population group for granted when identifying target populations for education and sensitisation programs on EVD or other infectious disease outbreaks. Third, health workers’ risk awareness and safety messages about condom use by male survivors following their re-integration into their communities need to be targeted exclusively at the affected individuals and couples, not at the wider community. This would guard against unintended stigmatisation of survivors and their families.

## Conclusion

EVD patients’/survivors’ experiences across the entire spectrum of their illness – from the period before diagnosis to their treatment at the EVD Treatment Unit and, later, their re-integration into their communities – are characterised with high levels of unattended psychological distress. We propose a holistic person-centred approach to healthcare for EVD suspects, confirmed cases and survivors. Additionally, EVD education and sensitisation programs need to target health workers, among other population groups.

## Supporting information

S1 Text Interview Guide. (DOCX)
