# Supplementary material for: A qualitative study of Ebola survivors’ psychological experiences of evacuation, treatment and community reintegration: Lessons in holistic person-centred care from the 2022 outbreak in Uganda
Source: PLOS Ment Health. 2025 Jun 23;2(6):e0000316. doi: 10.1371/journal.pmen.0000316 (PMC12798338; doi:10.1371/journal.pmen.0000316)
Supplement: S1 Text — (DOCX) [file pmen.0000316.s001.docx]

**A STUDY TO EXPLORE THE EVD ASSOCIATED NEGATIVE BELIEFS AND LIVED EXPERIENCES OF AFFECTED MEMBERS OF THE COMMUNITY**

**INTERVIEW GUIDE for EVD SURVIVORS**

**Introductions and building rapport; biodata (name, age, sex, religion, marital status, education level, etc.)**

As someone who has suffered from EVD, hearing from you would provide us with a rich understanding of what it means to be an EVD patient and survivor.

1. We would like you to tell us about your experience as an EVD patient – what was it like to suffer from EVD?

Probes:

- Experience before hospitalisation
- Experience in isolation (if applicable)
- Experience of symptoms
- Experience of evacuation
- Experience at the Treatment Unit?
- Were there any gaps in the clinical management of EVD patients like you?
- Treatment by health workers?
- Treatment by relatives?
- Treatment by community members?
- Any distressful experiences at the treatment unit or in your relationship with
  - health workers
  - relatives
  - community members
- If anything, what could have been done better while you were undergoing treatment?

1. What has it been like to be an EVD survivor (after being discharged from the EVD Treatment unit)?

Probes:

- Relationship with immediate and extended family
- Relationship with community members
- Any positive experiences in your relationship with immediate and extended family or community members?
- Any distressful experiences in your relationship with immediate and extended family or community members?
- (If any) How have you coped with these distressful experiences?
- Have you ever been stigmatised and discriminated by immediate and extended family or community members because of having suffered from EVD?
- (If yes) How have you coped with this stigmatisation?
- Has your gender in any way influenced your ability to manage these distressful experiences?
- What kind of support should be given to EVD survivors like you in managing these distressful experiences?

1. What were:

(a) your beliefs about causes of EVD at the time you were infected?

(b) the beliefs within your community about causes of EVD at the time you were infected?

Probes:

- Witchcraft?
- Other religious beliefs?
- Economic?
- Politics?

1. Did your beliefs or the beliefs within the community at the time you fell sick affect your choice of treatment seeking and timing, and if so, how?
2. Did your gender influence your choice of treatment seeking and timing, and if so, how?
3. Given your experience as an EVD survivor, and knowing what you know now, have your beliefs about the causes of EVD changed, and if so, explain?
4. Any other treatment options you had used before or after being discharged that you think have been helpful or unhelpful?

Probes: - Prayers

- Survivors’ clinic

- Traditional rituals/ceremonies

- Others
